# Experimental Investigation on the Influence of Depth on Rockburst Characteristics in Circular Tunnels

**DOI:** 10.3390/s22103679

**Published:** 2022-05-12

**Authors:** Xuefeng Si, Kang Peng, Song Luo

**Affiliations:** School of Resources and Safety Engineering, Central South University, Changsha 410083, China; xuefengsi@csu.edu.cn (X.S.); luosong@csu.edu.cn (S.L.)

**Keywords:** circular tunnel, rockburst, depth, true-triaxial test, V-shaped notch

## Abstract

To investigate the influence of depth on the rockburst of surrounding rock in a circular tunnel, true-triaxial tests at different depths were carried out on cubic granite specimens with a circular through-going hole. A micro camera was used to monitor the rockburst process of the circular hole sidewall in real time. The test results show that the failure process at different depths can be divided into four periods: the calm period, the particle ejection period, the rock fragment exfoliation period, and the rock bursting period. With an increase in depth, the three-dimensional unequal stress state gradually increased; the failure range and the size of rock fragments increased, the initial failure vertical stress linearly increased, and the strength and stability of the surrounding rock were enhanced. Therefore, the support range of surrounding rock should be increased as the depth increased to improve the overall stability of surrounding rock and reduce the failure range.

## 1. Introduction

To meet the development and needs of the country, many underground projects have appeared, and the depth has gradually increased [1,2,3,4,5]. The depth has a significant impact on the mechanical properties of the rock. For example, Peng et al. [6,7] conducted uniaxial compression tests and dynamic impact tests on granite from various burial depths, and obtained that peak stress was fitted with a quadratic function in terms of the change in burial depth. Wang et al. [8] reported that the uniaxial compressive strength and the rockburst proneness increased by carrying out uniaxial compression tests on different buried deep limestones. After the underground engineering enters the deep, the deep rock mass is in a complex environment such as high geostress, high ground temperature, high karst water pressure and strong mining disturbance [9,10,11], which leads to a series of deep disasters, and rockburst is one of the common major disasters [12,13]. Rockburst is accompanied by the ejection of rock fragments and the release of a large amount of energy [14,15,16,17,18,19]. Strong rockburst often brings catastrophic consequences, causing casualties and damage to construction equipment, seriously hindering the smooth progress of production [20,21,22]. For example, on 31 May 2015, an extremely intense rockburst struck the headrace tunnel of the Neelum–Jhelum hydropower station in Pakistan, causing 3 deaths, 17 injuries, and serious damage to the TBM and surrounding rock mass. Consequently, more than half a year was needed for recovery before construction could continue [23,24]. On 28 November 2009, an extremely strong rockburst in a drainage tunnel at a depth of 2330 m caused seven deaths and one injury as well as the total destruction of the tunnel boring machine [25,26]. Since 2016, the mining depth of Yunnan Dahongshan Iron Mine has gradually entered the range of 767–1261 m, and the maximum development depth has reached 1301 m. During the construction process, several slight and moderate rockbursts occurred, and the number of rockbursts increased exponentially with the increase in burial depth [27]. Therefore, with the increase in depth, rockburst disaster becomes more prominent [28,29,30,31,32].

In view of the hazards of rockburst disasters, many scholars conducted a series of studies on the rockburst process and occurrence mechanism [33,34,35,36,37,38,39,40]. Singh [33,34] proposed that the use of burst proneness index to analyze rockburst was a simple and reliable method, and the physical and mechanical properties under uniaxial compression had an important influence on the burst proneness index. Si et al. [35,36] reported that the influence of bedding angle and loading rate on the rockburst process and characteristics under the uniaxial and triaxial compression. Wu et al. [37] obtained that the number of openings had a significant impact on the weakening effect of rock mechanical properties, and explained the crack initiation mechanism under the uniaxial compression. Hu et al. [38] observed the rockburst process of the surrounding rock in circular holes under two-dimensional stress conditions, and analyzed the spatial distribution and structural response characteristics of the rockburst process. Gong et al. [39,40] obtained that the effect of lateral stress on rockburst was more obvious than that of horizontal axial stress, and increasing the lateral stress can significantly reduce the severity of rockburst by conducting the true-triaxial tests on cube samples of 100 mm × 100 mm × 100 mm. However, there are relatively few studies on the influence of depth on the rockburst. As the depth increased, the elastic strain energy stored in the rock mass increased, and more elastic strain energy was released when rock failure occurred [41,42]. Therefore, it is necessary to carry out experimental studies on the effect of depth on rockburst process, characteristics and severity of the surrounding rock of the circular tunnel.

In this study, true-triaxial tests were conducted using a true-triaxial testing machine on 100 mm cubic granite specimens containing a circular through-going hole at different depths. The true-triaxial tests were carried out in strict accordance with the testing procedure in the ISRM Suggested Method (determining deformation and failure characteristics of rocks subjected to true triaxial compression) [43]. During the tests, the rockburst process of hole sidewall was monitored and recorded in real time by a micro camera. The influence of depth on rockburst characteristics and severity in circular hole was investigated. This study has important guiding significance for the prevention and control of surrounding rock in circular tunnel at different depths.

## 2. Experimental Procedures

### 2.1. Sample Preparation

**Figure 1:**
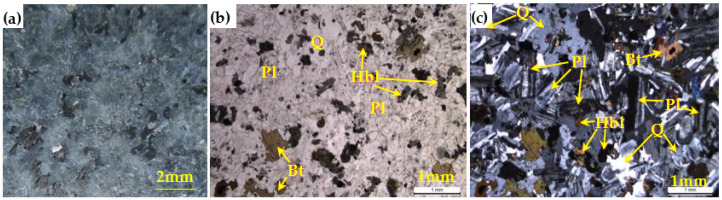
Granite material: (**a**) naked-eye observation, (**b**) plane-polarized light, and (**c**) cross-polarized light (the letters Q, Pl, Bt, and Hbl represent quartz, plagioclase, biotite, and hornblende, respectively).

The granite material was obtained from Changtai County, Zhangzhou City, Fujian Province, China. The naked-eye observation photo of granite material is shown in Figure 1a. A thin rock section was observed with a petrographic microscope under plane-polarized and cross-polarized lights, as shown in Figure 1b,c, and the mineral composition of the rock was approximately 49% quartz, 43% plagioclase, 4% biotite, 3% hornblende, and 1% opaque minerals. Based on IUGS classifications [44], the selected rock material belongs to the granodiorite.

**Figure 2:**
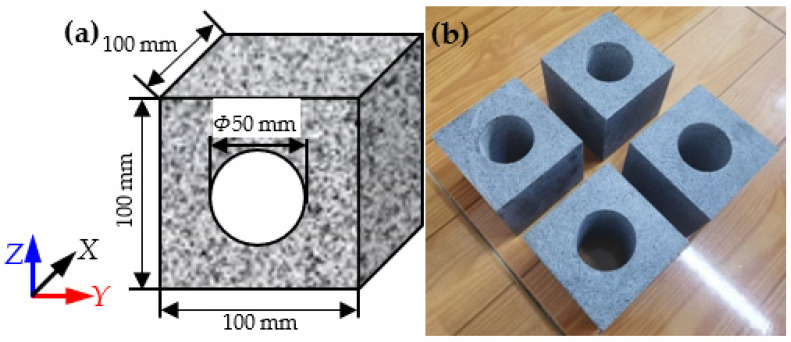
Granite specimens: (**a**) schematic of the specimen, (**b**) image of specimens.

The granite material was cut into cubes with side lengths of 100 mm, and then a cylindrical through-going hole with a diameter of 50 mm was drilled in the center of the specimen, as shown in Figure 2. The machining precision strictly followed the ISRM standards for the cubic samples [43]. The P-wave velocity and density of the granite material were 4.9 km/s and 2766 kg/m^3^; the uniaxial compressive strength (UCS), elastic modulus, and Poisson’s ratio of granite material were 261.6 MPa, 57.9 GPa, and 0.21, respectively. The granite material in this study had strong rockburst proneness [45,46].

### 2.2. True-Triaxial Testing Machine

**Figure 3:**
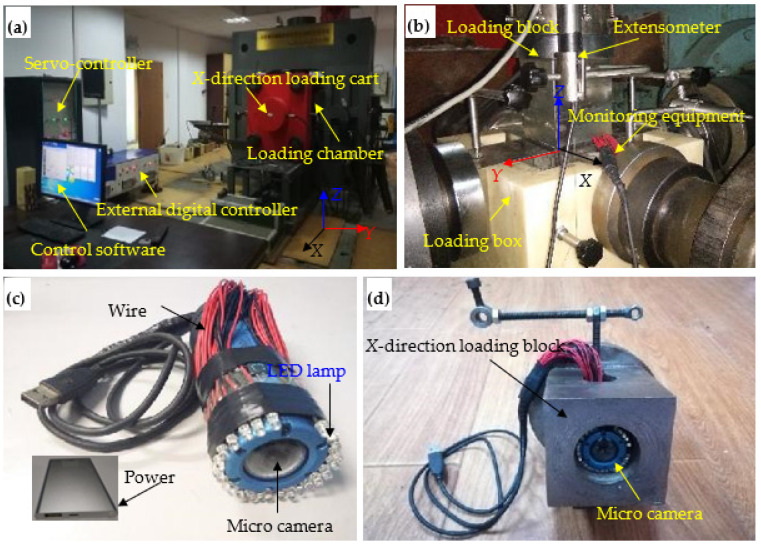
Experimental equipment: (**a**) true-triaxial testing machine, (**b**) loading chamber, (**c**) microcamera, and (**d**) combination of microcamera and *X*-direction loading block.

A true-triaxial testing system (Figure 3a,b) was used during the tests, which was introduced in detail in Gong et al. [39,40]. A micro camera (Figure 3c) was used to monitor the failure process of the circular hole sidewall in real time. The combination of the micro camera and the *X*-direction loading block is shown in Figure 3d. The stress monitoring system is composed of a true-triaxial frame, a servo controller, an external digital controller, a force sensor, a data acquisition instrument, a computer and a rock true-triaxial servo control software. The true-triaxial frame and the loading blocks are composed of high-rigidity metal materials, which have high compressive strength, tensile strength and strong resistance to deformation [47,48,49]. During the test, the experimenter transmits load command to be applied to the servo controller through the computer and rock true-triaxial servo control software, and then it controls the oil pump to apply the corresponding oil pressure, which pushes the loading rod to act on the loading block, and finally realizes sample loading. The force loaded on the sample is transmitted to the external digital controller through the force sensor on the loading rod collected by the data acquisition instrument, and the final test curve is presented on the control software.

### 2.3. Initial Stress States at Different Depths

**Figure 4:**
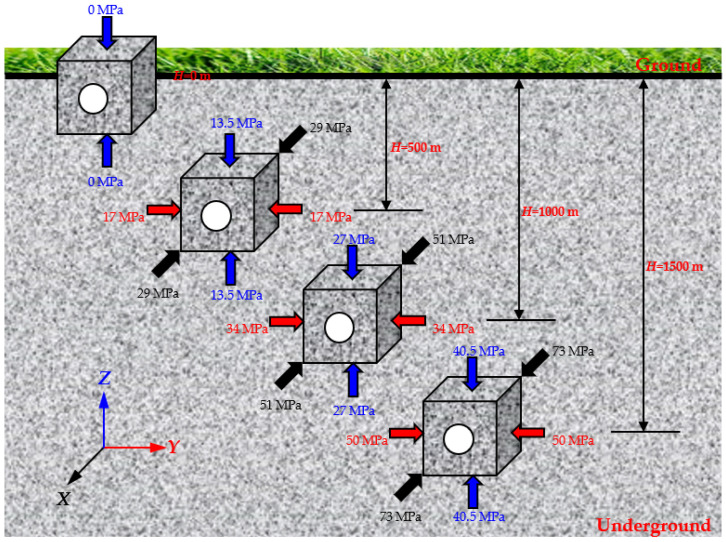
Initial stress states at the depths of 0, 500, 1000, and 1500 m.

At the depths of 0, 500, 1000, and 1500 m, the initial stress state was determined by the in situ stress calculation Formula (1) [50,51].

(1) $$\left.\begin{array}{l} \sigma_{v}=0.027H\\ \sigma_{\mathrm{hmax}}=6.7+0.044\;4H\\ \sigma_{\mathrm{hmin}}=0.8+0.032\;9H \end{array}\right\}$$

where $\sigma_{v}$ is the vertical stress (MPa), $\sigma_{\mathrm{hmax}}$ and $\sigma_{\mathrm{hmin}}$ are the maximum and minimum horizontal stresses (MPa), respectively, *H* is the depth (m). In the underground rock mass, most of the regions have three-dimensional unequal compressive stress dominated by the vertical and horizontal stresses. Therefore, the initial stress field of three-dimensional unequal compressive stress is set according to the calculations of in situ stress at different depths, as shown in Figure 4. According to the experimental results from Gong et al. [39,40], when the lateral stress is higher, the failure stress of the circular hole sidewall is higher, and the stability of surrounding rock is stronger. The uniaxial compressive strength of the granite selected in this study reaches 261.6 MPa. Under three-dimensional stress, the initial failure stress of surrounding rock is relatively high. However, due to the limitation of stress range of the testing machine, the surrounding rock is difficult to be significantly damaged under high lateral stress. To make the test results more obvious, this study mainly carried out the true-triaxial test in which the *X* direction stress at different depths serves as the horizontal maximum principal stress *σ*_hmax_.

## 3. Experimental Results

### 3.1. Stress Path and Stress–Time Curves

**Figure 5:**
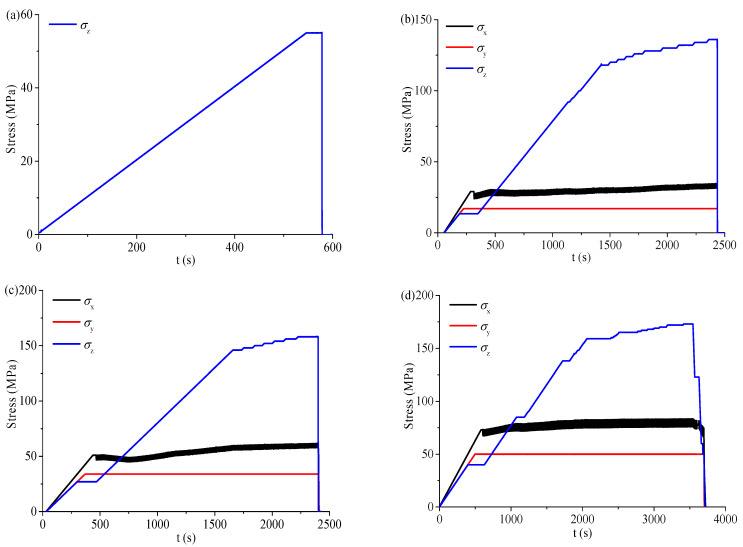
Stress–time curves at different depths: (**a**) *H* = 0 m, (**b**) *H* = 500 m, (**c**) *H* = 1000 m, and (**d**) *H* = 1500 m.

During the test, *σ*_x_, *σ*_y_, and *σ*_z_ were increased to the initial stress state under different depths by load control at the loading rate of 1000 N/s. Then, the loading method in the *X* direction was changed to displacement control, that is, the displacement in the *X* direction remained unchanged (simulated plane-strain problem). *σ*_z_ continued to increase at the same loading rate. When serious spalling failure occurred on both sidewalls of the circular hole, *σ*_z_ was maintained for a period, and then *σ*_x_, *σ*_y_, and *σ*_z_ were decreased to 0 MPa. The stress–time curves at different depths are shown in Figure 5. At the depth of 0 m, the shallow tunnel was simulated, and the horizontal stress was 0 MPa, as shown in Figure 5a. At the depths of 500, 1000, and 1500 m, the initial stress states (*σ*_x_, *σ*_y_, *σ*_z_) were (29, 17, 13.5), (51, 34, 27), and (73, 50, 40.5), respectively. When *σ*_x_, *σ*_y_, and *σ*_z_ were increased to the initial stress states by load control, the loading method in the X-direction was changed from force to displacement control, and *σ*_x_ produced slight fluctuation, as shown in Figure 5b–d. When *σ*_z_ was increased to 55 MPa, 136 MPa, 158 MPa, and 173 MPa at the depths of 0, 500, 1000, and 1500 m, *σ*_x_, *σ*_y_, and *σ*_z_ were decreased to 0 MPa at the displacement rate of 20 mm/min.

### 3.2. Failure Process of the Circular Hole Sidewall

**Figure 6:**
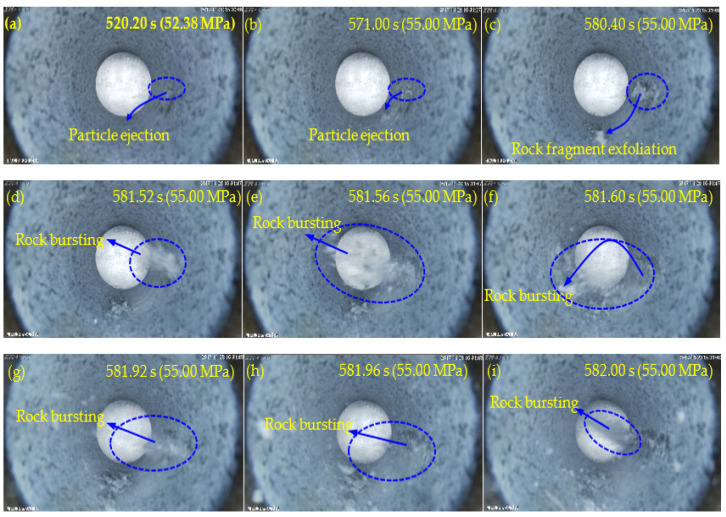
Failure process at *H* = 0 m.

**Figure 7:**
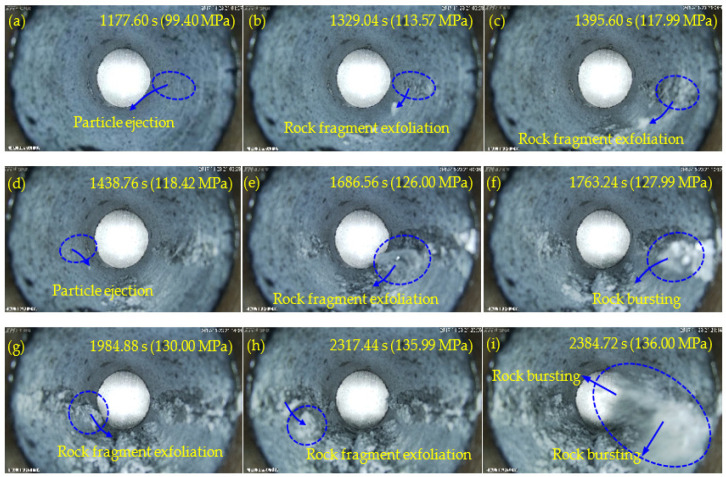
Failure process at *H* = 500 m.

**Figure 8:**
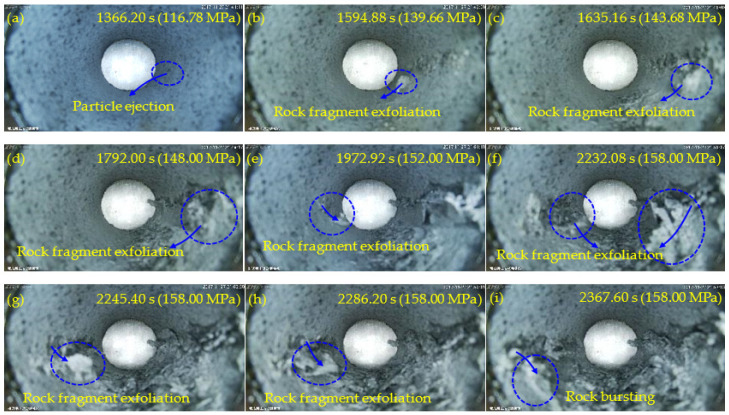
Failure process at *H* = 1000 m.

**Figure 9:**
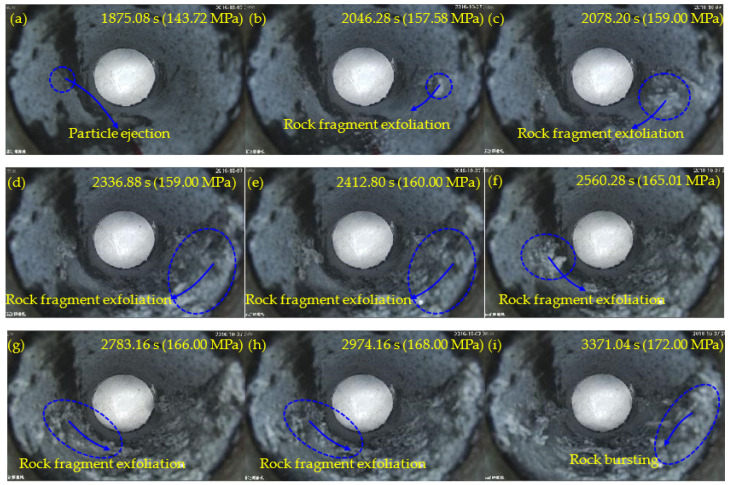
Failure process at *H* = 1500 m.

**Figure 10:**
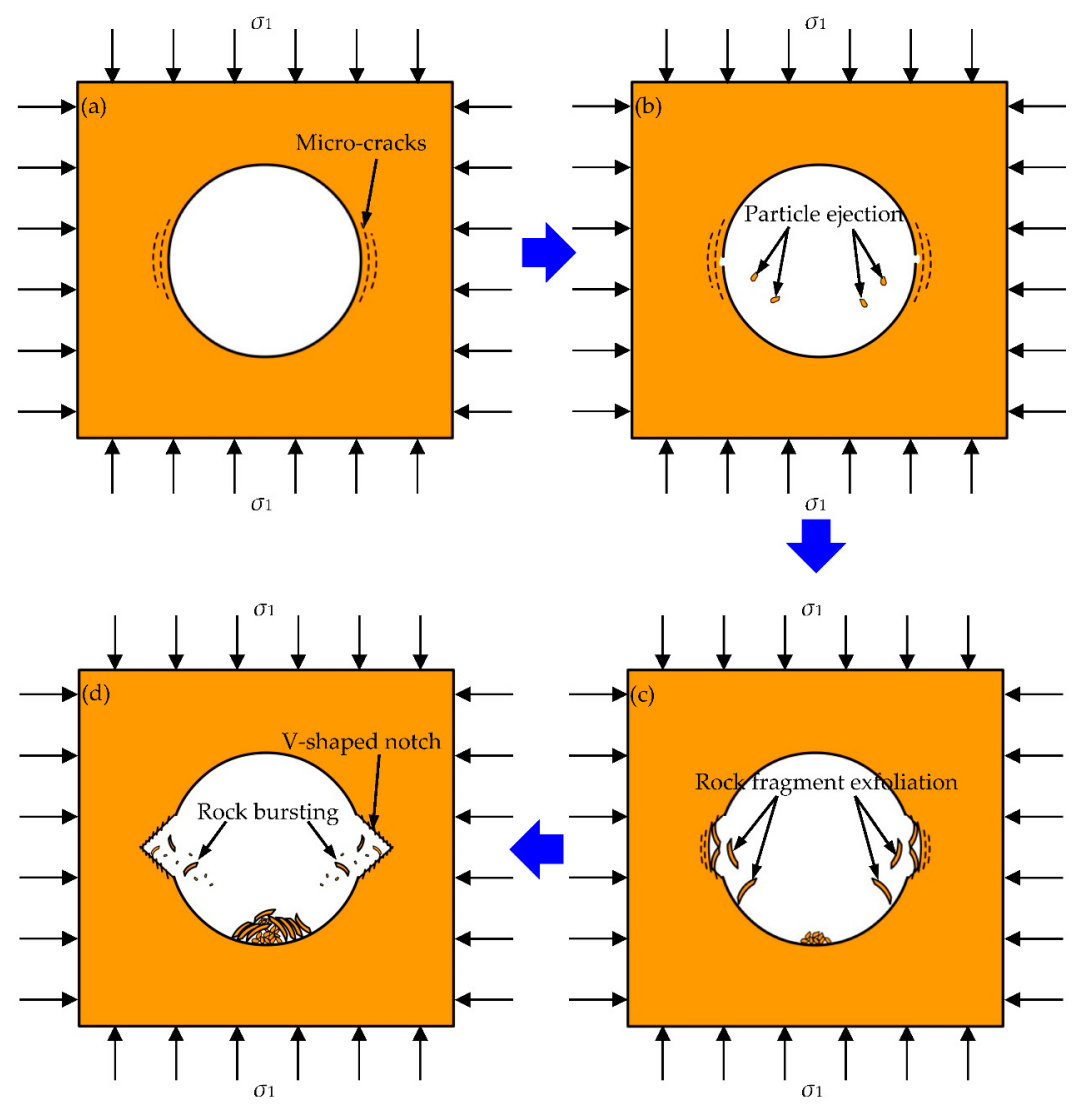
Schematic diagrams of the four periods of sidewall failure: (**a**) the calm period, (**b**) the particle ejection period, (**c**) the rock fragment exfoliation period, and (**d**) the rock bursting period [40].

Figure 6, Figure 7, Figure 8 and Figure 9 show the failure processes of the circular hole sidewalls at the depths of 0, 500, 1000, and 1500 m. When *H* = 0 m, the failure process of sample HK-0-0 is shown in Figure 6. At 520.20 s and when *σ*_z_ = 52.38 MPa, the right sidewall experienced particle ejection, as shown in Figure 6a. When *σ*_z_ was increased to 55.00 MPa and maintained constant, the right sidewall produced particle ejection (Figure 6b), rock fragment exfoliation (Figure 6c), and multiple violent failures (Figure 6d–i). During the violent failure, the right sidewall suffered massive particle ejection and rock fragment exfoliation, as shown in Figure 6d–i. When *H* = 500 m, the confining pressure was increased to 29 MPa (*σ*_x_) and 17 MPa (*σ*_y_). The failure process of sample HK-29-17 is shown in Figure 7. During loading to the initial stress state, no macroscopic failure occurred on the circular hole sidewalls. At 1177.60 s, *σ*_z_ was increased to 99.40 MPa, and fine particle ejection occurred on the right sidewall, as shown in Figure 7a. When *σ*_z_ = 113.57 MPa, the right sidewall produced rock fragment exfoliation (Figure 7b). As *σ*_z_ was continuously increased to 117.99 MPa, the right sidewall produced rock fragment exfoliation (Figure 7c). When *σ*_z_ was loaded to 118.42 MPa, the left sidewall experienced fine particle ejection, as shown in Figure 7d. In Figure 7e, large rock fragment was exfoliated and accompanied by massive particle ejection when *σ*_z_ was increased to 126.00 MPa. As shown in Figure 7f, when *σ*_z_ = 127.99 MPa, the right sidewall produced rock bursting accompanied by massive particle ejection and rock fragment exfoliation. With an increase in *σ*_z_, two rock fragment exfoliations occurred on the left sidewall, as shown in Figure 7g,h. Finally, when *σ*_z_ = 136.00 MPa, the right sidewall produced rock bursting, and ejected massive particles and rock fragments, as shown in Figure 7i. At the depths of 1000 and 1500 m, the failure processes (Figure 8 and Figure 9) of the circular hole sidewalls were similar to that at the depths of 0 and 500 m. In summary, the failure process at different depths can be divided into four periods: (a) the calm period, (b) the particle ejection period, (c) the rock fragment exfoliation period, and (d) the rock bursting period. The schematic diagrams of the failure process at different stages are shown in Figure 10. With expansion of failure depth, symmetrical V-shaped notches were finally formed on both sidewalls perpendicular to the direction of the maximum principal stress.

### 3.3. Failure Characteristics of the Circular Hole Sidewall

**Figure 11:**
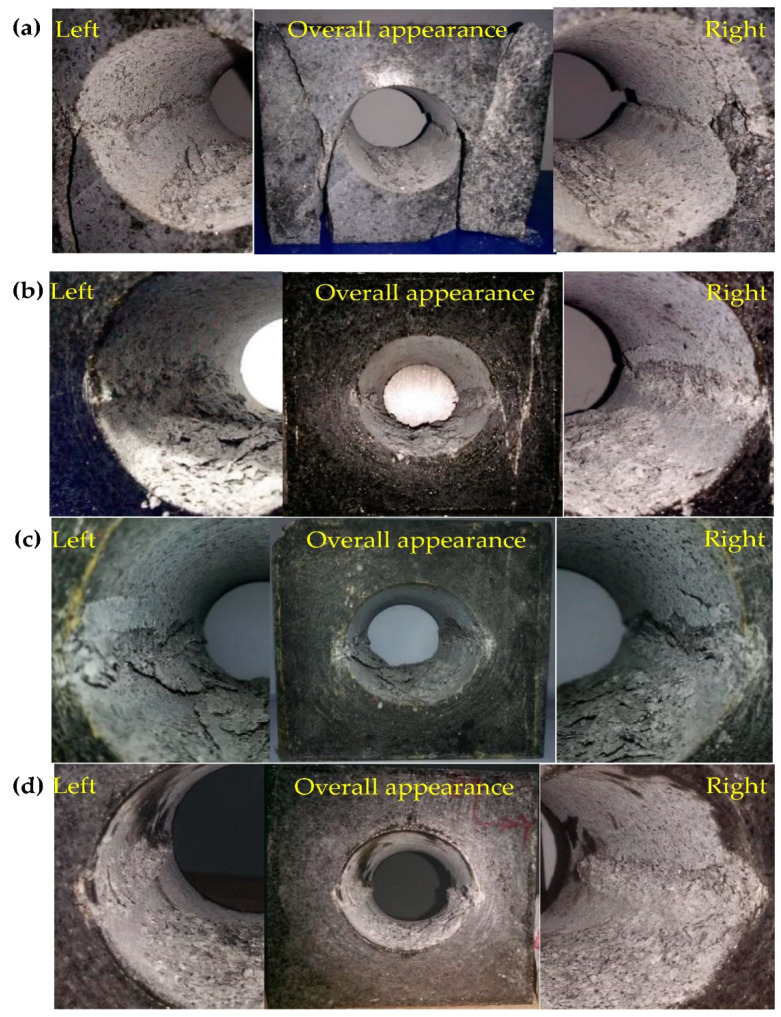
Photographs of failure at different depths: (**a**) *H* = 0 m, (**b**) *H* = 500 m, (**c**) *H* = 1000 m, and (**d**) *H* = 1500 m.

The left and right sidewall failures at different depths are shown in Figure 11. The failure positions of the granite tunnel at the depths of 0, 500, 1000, and 1500 m were distributed in the sidewalls perpendicular to the direction of the maximum principal stress, and symmetrical V-shaped notches were formed on both sidewalls with the increase in the maximum principal stress (vertical stress, *σ*_z_). During the sidewall failure, the ejected particles and exfoliated rock fragments had a certain speed, indicating that the energy accumulated in the surrounding rock partially transformed the kinetic energy of the rock fragments. From Figure 11, the rock fragments produced by the sidewall failure were mostly distributed at the bottom of the circular hole. Therefore, when the vertical stress is the maximum principal stress, the depth has no effect on the failure position of the surrounding rock in circular hole.

**Figure 12:**
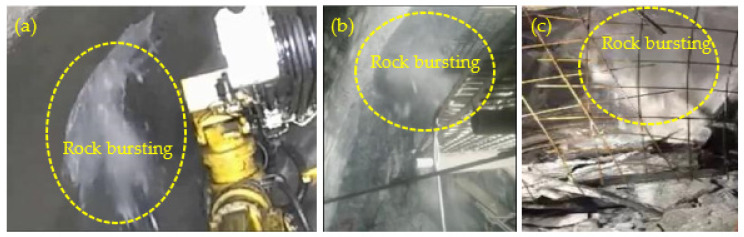
Photographs of rockburst in the actual engineering: (**a**) Bayu Tunnel in Lalin Section of Sichuan–Tibet Railway, (**b**) Grand Canyon Tunnel of Sichuan ehan Expressway, and (**c**) Qinling Tunnel for the water diversion from the Han to Wei river project.

Figure 12 shows the rockburst occurred in the actual engineering. For example, for Bayu Tunnel in Lalin Section of Sichuan–Tibet Railway (Figure 12a), Grand Canyon Tunnel of Sichuan ehan Expressway (Figure 12b), and Qinling Tunnel for the water diversion from the Han to the Wei river project (Figure 12c), the surrounding rock of these tunnels suffered from severe rockburst, and the rock bursting processes were accompanied by massive particle ejection and rock fragment exfoliation. This is consistent with the experimental results in Section 3.2, as shown in Figure 6d–i, Figure 7f,i, Figure 8i and Figure 9i. Therefore, the laboratory simulation tests reproduced the rockburst process of the surrounding rock in a circular tunnel at different depths, and the test results were relatively reliable.

## 4. Discussions

### 4.1. Influence of Depth on Failure Characteristics

**Figure 13:**
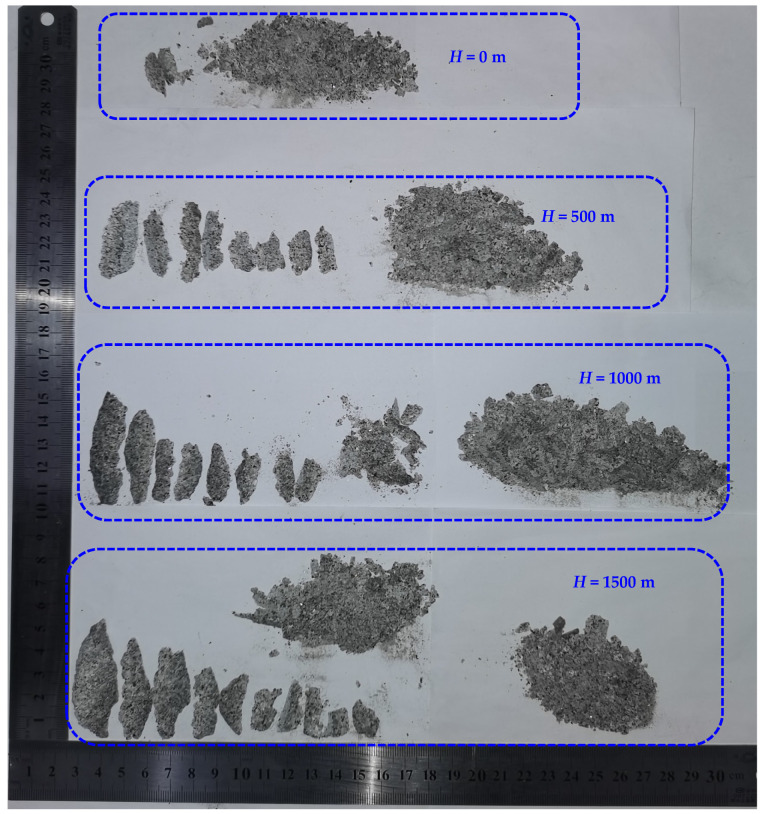
Rock fragments produced by the circular hole sidewall at different depths.

Figure 13 shows the rock fragments produced by the circular hole sidewalls at different depths. When the depth was 0 m, the sidewall failure produced mostly fine rock particles, and the failure range of the left and right sidewalls was relatively small. When *H* = 500 m, the failure of the left and right sidewalls mostly produced rock fragments showing the characteristics of thick in the middle and thin at the edge. During the rock fragment exfoliation, the sidewall failure also produced some fine particles. By comparison, the failure range at the depth of 500 m was obviously wider than that when *H* = 0 m, and the size of rock fragments was larger, as shown in Figure 11 and Figure 13. When *H* = 1000 m, the size of rock fragments further increased and was close to that at the depth of 1500 m. Therefore, within a certain depth range, with increasing depth, the failure range of circular tunnel increased, and the size of rock fragments increased. However, the damage range does not always increase with the depth, and there may be a critical value. Since only four depths were carried out in this study, more experimental studies are needed to determine the critical depth.

**Figure 14:**
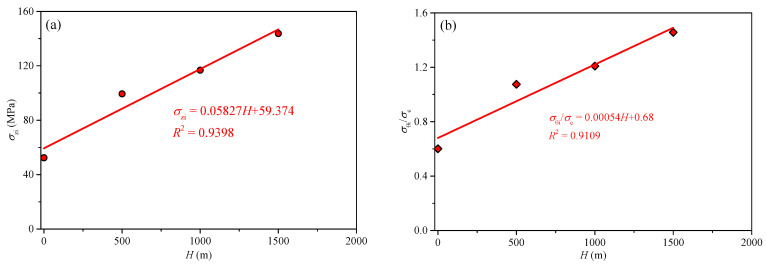
Stress characteristics of initial sidewall failure at different depths: (**a**) relationship between *σ*_zi_ and *H*; (**b**) relationship between *σ*_θi_/*σ*_c_ and *H*.

Figure 14a shows the initial failure vertical stress *σ*_zi_ of circular hole sidewalls at different depths. It shows that *σ*_zi_ increased linearly with the increase in depth. The main reason was that the three-dimensional unequal stress state of the surrounding rock gradually increased with the increase in depth, which in turn led to an increase in the strength and stability of the surrounding rock. Therefore, the fundamental reason for the change in surrounding rock stability at different depths is the change in the stress state. To analyze stress distribution in the surrounding rock of a circular tunnel, it is assumed that the rock mass before failure is elastic. According to Kirsch solution, the maximum tangential stress *σ*_θi_ is distributed on the left and right sidewalls. Therefore, results of *σ*_θi_ = 3*σ*_zi__−_*σ*_y_, and *σ*_θi_/*σ*_c_ (*σ*_c_ is the uniaxial compressive strength) can be obtained and listed in Table 1. When the sidewall produced initial failure, the range of *σ*_θi_/*σ*_c_ was from 0.60 to 1.46 at the depths of 0, 500, 1000, and 1500 m, as shown in Figure 14b. The test results agree with those of Dowding and Andersson [52]. From Figure 14b, it is noted that the *σ*_θi_/*σ*_c_ increases linearly with depth.

### 4.2. Influence of Depth on Rockburst Severity

**Figure 15:**
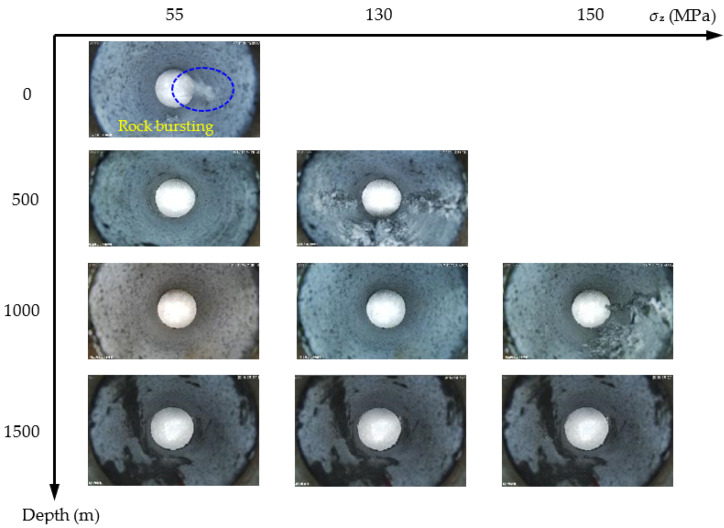
Failure of the circular hole sidewall under different depths and vertical stresses.

To compare the failure severity of surrounding rock in the circular tunnel at different depths, the failure photographs of the sidewalls were selected when the vertical stress was adjusted to 55, 130, and 150 MPa, as shown in Figure 15. When *σ*_z_ = 55 MPa, a violent rockburst occurred on the circular hole sidewall, and the right sidewall produced massive particle ejection and rock fragment exfoliation at the depth of 0 m. At the depths of 500, 1000, and 1500 m, there was no macroscopic failure on the surrounding rock. By comparison, the rockburst of the surrounding rock in the circular tunnel was the most severe at the depth of 0 m. Since there was no confinement effect of confining pressure at the depth of 0 m, the bearing capacity of the specimen was low, and the vertical stress was below 130 MPa. Therefore, when *σ*_z_ = 130 MPa, the failure photographs of the sidewalls at the depths of 500, 1000 and 1500 m were taken. The left and right sidewalls at the depth of 500 m produced severe failure, and cracks approximately penetrated the entire sample along the axis of the circular hole. At the depth of 1000 m, slight particle ejection occurred on the right sidewall, but no macroscopic failure occurred on the left sidewall. At the depth of 1500 m, no macroscopic failure occurred on the surrounding rock. Since the confining pressure at the depth of 500 m was lower than that at the depths of 1000 and 1500 m, the bearing capacity of the specimen was lower, and the failure at the depth of 500 m was more severe than that at the depths of 1000 and 1500 m. Finally, the vertical stress was not increased to 150 MPa. Therefore, when *σ*_z_ = 150 MPa, the failure photographs of the sidewalls at the depths of 1000 and 1500 m were taken. Severe failure occurred on the right sidewall at the depth of 1000 m, in which cracks approximately penetrated the entire sample along the axis of the circular hole. At a depth of 1500 m, slight particle ejection occurred on the left and right sidewalls in the circular tunnel. By comparison, the failure at the depth of 1000 m was significantly more severe than that at the depth of 1500 m. In summary, when the vertical stress was adjusted to the same stress level, with the increase in depth, the failure severity of the surrounding rock in the circular tunnel gradually decreased. However, when the vertical stress was adjusted to a higher stress level, the failure range of the surrounding rock of the circular tunnel was wider with the increase in depth. Therefore, the support range of the surrounding rock should be increased as the depth increased to improve the overall stability of the surrounding rock and reduce the failure range.

### 4.3. Reliability and Repeatability of Test Results

Generally, to ensure the accuracy and reliability of experimental results, three tests should be conducted under the same state. However, due to the limited number of granite samples collected from the site, only four cube samples with a circular hole were left and prepared for tests of this paper. To study the influence of depth on the failure process and characteristics of the surrounding rock in a circular tunnel, we carried out experiments at four depths. Due to the limited number of samples, we set the lateral stress as the minimum horizontal principal stress and the hole axis stress as the maximum horizontal principal stress at each depth. Then, true-triaxial tests were conducted at different depths. We illustrate the reliability and repeatability of the experimental results in this study from the following aspects.

**Figure 16:**
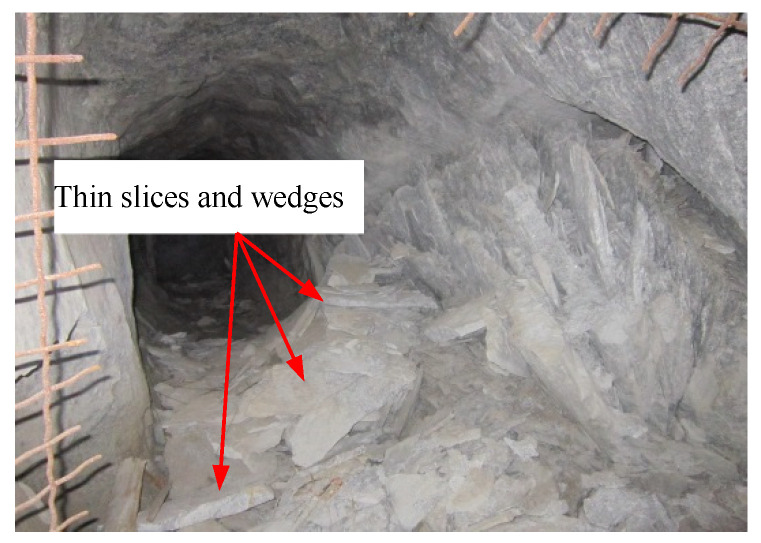
Rock fragments in a transport roadway at 500 m in Linglong gold mine, China.

(1) At different depths, although the stress state of the surrounding rock has changed, the failure process of the surrounding rock is similar and can be divided into four stages: (a) the calm period, (b) the particle ejection period, (c) the rock fragment exfoliation period, (d) the rock bursting period. Therefore, the results show the repeatability and reliability of the failure process of the circular tunnel surrounding rock in this manuscript.

(2) After the tests, V-shaped notches were formed on the left and right sidewalls in the circular tunnel (Figure 11), which is similar to the failure characteristics in the actual project. For example, spalling in the roof and floor of a circular test tunnel in the underground research laboratory [53], and mild spalling in the sidewalls of a vertical raise bored shaft in an underground mine [54]. This indicates that the test results are consistent with the actual project.

(3) The rock fragments produced by the failure of the tunnel sidewall were mostly thin slices and wedges (Figure 13), which were similar to that produced by rockbursts in a transport roadway at 500 m in Linglong gold mine (Figure 16).

(4) According to the test results, with the increase in depth, the three-dimensional unequal stress state of the surrounding rock gradually increased; the failure range and the size of rock fragments increased, and the initial failure vertical stress linearly increased, the strength and stability of the surrounding rock were enhanced. Therefore, the fundamental reason for the change in surrounding rock stability at different depths is the change in the stress state. The test results did not show abnormal data, so the test results are more reliable.

In summary, although only one experiment is carried out for each working condition in this study, the test results are repeatable and reliable.

## 5. Conclusions

(1) The failure process at different depths can be divided into four periods: (a) the calm period, (b) the particle ejection period, (c) the rock fragment exfoliation period, and (d) the rock bursting period. With expansion of the failure depth, symmetrical V-shaped notches were finally formed on both sidewalls perpendicular to the direction of the maximum principal stress. The failure characteristics were consistent with the spalling failure observed in underground engineering.

(2) With an increase in depth, the three-dimensional unequal stress state of the surrounding rock gradually increased, the failure range and the size of rock fragments increased, the initial failure vertical stress linearly increased, and the strength and stability of the surrounding rock were enhanced. Therefore, the fundamental reason for the change in surrounding rock stability at different depths is the change in the stress state.

(3) When the vertical stress was adjusted to the same stress level, with an increase in depth, the failure severity of surrounding rock in the circular tunnel gradually decreased. However, when the vertical stress was adjusted to a higher stress level, the failure range was wider with increasing depth. Therefore, the support range of the surrounding rock should be increased as the depth increased to improve the overall stability of the surrounding rock and reduce the failure range.

## Figures and Tables

**Table 1:** Stress characteristics of initial sidewall failure at different depths.

*H* (m)	*σ*_y_ (MPa)	*σ*_zi_ (MPa)	*σ*_θi_ (MPa)	*σ*_θi_/*σ*_c_
0	0	52.4	157.2	0.60
500	17	99.4	281.2	1.07
100	34	116.8	316.4	1.21
1500	50	143.7	381.1	1.46

## Data Availability

All data generated or analyzed during this study are included in this published article.
